# Synchronization of passive interpersonal light touch and body control responses during walking

**DOI:** 10.1186/s12984-025-01799-2

**Published:** 2025-12-03

**Authors:** Tsubasa Mitsutake, Hisato Nakazono, Takanori Taniguchi, Hisayoshi Yoshizuka, Maiko Sakamoto

**Affiliations:** 1 Clinical Research Center, Saga University Hospital, 5-1-1 Nabeshima, Saga, 849-8501 Japan; 2 Department of Occupational Therapy, Faculty of Medical Science, Fukuoka International University of Health and Welfare, 3-6-40 Momochihama, Sawara-ku, Fukuoka, 814-0001 Japan; 3 Department of Physical Therapy, Faculty of Medical Science, Fukuoka International University of Health and Welfare, 3-6-40 Momochihama, Sawara-ku, Fukuoka, 814-0001 Japan; 4 Education and Research Centre for Community Medicine, Faculty of Medicine, Saga University, 5-1-1 Nabeshima, Saga, 849-8501 Japan

**Keywords:** Passive interpersonal light touch, Walking support, Contact provider, Contact receiver, Phase locking value, Root mean square, Harmonic ratio, Autocorrelation coefficient

## Abstract

**Background:**

Passive interpersonal light touch (PILT) provides postural coordination cues to the body of a contact receiver through the fingertips of a contact provider. However, the manner in which the fingertip information of the PILT contact provider affects the gait performance of the contact receiver remains unclear. We aimed to investigate body control responses such as trunk sway and smoothness to PILT during walking to clarify the synchronization of fingertip–body movements in the context of the experience the PILT provider in body manipulation.

**Methods:**

In total, 17 healthy volunteers (21.4 ± 1.1 years old, 9 females) performed 10-m walk tests under three conditions: walking without touch (NT), walking with PILT administered by contact provider with extensive rehabilitation experience in manual body manipulation (PILT_E_), and walking with PILT administered by contact provider with no experience (PILT_N_). A force sensor on the middle finger of the contact provider and inertial sensors on the third lumbar and seventh cervical spinous processes of the contact receiver were used to gather data pertaining to contact force, direction of motion, walking speed, triaxial root mean square, harmonic ratio (HR), autocorrelation, and phase locking value (PLV) to estimate the synchronization between the inertial and fingertip sensors for each condition.

**Results:**

The PILT_E_ group showed significantly higher walking speed and HR values in the anteroposterior (AP) direction than those of the NT and PILT_N_ groups. Additionally, the PLVs were significantly higher in the mediolateral (ML) direction in the PILT_E_ group than in the PILT_N_ group. In contrast, applying PILT_E_ significantly reduced the PLVs in the AP direction compared with that observed for PILT_N_.

**Conclusions:**

These findings suggest that the fingertips of skilled contact provider and the shift in the center of gravity of the contact receiver synchronize in the ML direction, which affects walking speed and smooth movement of walking patterns because of increased HR in the AP direction. Additionally, physical responses during walking to PILT administered by contact providers may change depending on the experience of the contact providers. Thus, the quantification of touch skills may potentially aid in administering effective walking assistance.

**Supplementary Information:**

The online version contains supplementary material available at 10.1186/s12984-025-01799-2.

## Background

Light touch (LT) involves the exertion of intentional contact with a force of < 1 N to stabilize postural retention [1, 2]. LT modulates spontaneous body sway by inducing postural coordination with both fixed objects and interpersonal objects. In the context of interpersonal LT, active interpersonal LT is administered to achieve postural stability by constructing spatial coordinate axes based on contact points made by the participant’s own fingertips, whereas passive interpersonal LT (PILT) provides cues for postural coordination to the body of the contact receiver through the fingertips of the contact provider. The use of PILT during walking increases the walking speed of the contact receiver and improves postural stability [3]. PILT is advantageous as it can be easily administered at any location as a method of assistance for general mobility-related movements without needing any equipment. PILT may be used as a training method because it affords control over the level of assistance provided by the contact provider. Therefore, understanding PILT-induced physical responses would provide novel information to support efficient mobile movement.

The benefit of PILT application during walking extends beyond merely providing support through touch because it potentially controls posture by effectively inducing center-of-gravity shifts while taking individual body characteristics into account. Additionally, interpersonal LT connects the provider and receiver and synchronizes interpersonal movements by defining the contact site as a spatial reference [4]. Therefore, the physical manipulation techniques used by the contact provider may affect the gait stability of the receiver. However, the effect of the direction of PILT movement instigated through the fingertip of the PILT contact provider on the gait performance of the contact receiver remains unclear. Healthcare workers who routinely provide mobility assistance may have developed body guidance techniques using PILT. Therefore, investigating the differences in the response (such as trunk sway and smoothness) to physical control exhibited by the contact receiver to contact providers with varying extent of experience may help elucidate the underlying PILT control mechanism.

Validating the PILT control mechanism requires the quantitation of the manner in which pressure is exerted through the fingertip of the contact provider and body movement occurs for the contact receiver. The skill of the provider may be quantitatively evaluated in terms of the direction of movement related to contact force using a small high-precision fingertip force sensor (FFS), which is a wearable unobtrusive device that acquires information from fingertips without restricting movement. The ability of the contact receiver to control posture while walking may be measured in detail using the inertial measurement unit (IMU). Simultaneous measurement using FFS and IMU is beneficial for evaluating synchrony, which enables the estimation of phase synchronization in each moving direction, and is advantageous for evaluating contact force and body movement synchronization. As LT induces interpersonal postural coordination [4], PILT may synchronize the fingertip–body response. Phase locking value (PLV) estimation is a useful technique for studying synchronization through the analysis of the homogeneous distribution of the relative phase for two waveforms of the same duration [5, 6]. Although PLV has been used to estimate the phase synchronization between transcranial alternating current electrical signals and postural control responses [7], it has not been used to estimate the phase synchronization between fingertips and body sway in response to PILT. To the best of our knowledge, this study is the first to attempt to apply this analysis to PILT feedback technique.

The purpose of this study was to use FFS and IMU to analyze the physical control responses of contact receivers during walking with PILT. Additionally, we aimed to investigate the change in synchronization between the directions of the contact provider’s fingertip movement and the contact receiver’s body control response based on the experience of the provider assisting in walking. We hypothesized that applying PILT would improve walking speed with high synchronization. Additionally, we hypothesized that the receiver’s gait would be better stabilized with an experienced rather than inexperienced provider.

## Methods

### Participants

In total, 18 healthy individuals with no history of neurological or orthopedic diseases that could affect their walking participated in this study. The appropriate sample size was estimated before starting the experiment using G Power version 3.1.9.7 [8] with the following assumptions based on a previous study: effect size (Cohen’s d), 1.007; α, 0.05; and power value (1-β error probability), 95% [3]. All experiments had a prospective design and were single-blind to examiners. Of the 18 participants, one was excluded because of missing values in the logged data. Hence, all data analyses included those of 17 participants. We recorded the assigned sex at birth for all participants as well as the self-identified gender; the data obtained did not differ for either categories for any of the participants.

The study was approved by the Ethics Review Committee (24-TG-007) and was conducted in accordance with the Declaration of Helsinki and its guidelines. All participants provided written informed consent after receiving an explanation of the study and its purpose.

### Experimental procedures

The experimental design was based on that of a previous study [3]. Gait measurements were performed under three scenarios: (1) PILT administered by a contact provider with extensive rehabilitation experience in manual body manipulation (PILT_E_); (2) PILT administered by a contact provider with no experience (PILT_N_); and (3) no-touch (NT). The contact provider in the PILT_E_ group was a skilled physical therapist with >10 years of experience in assisted activities, whereas the one in the PILT_N_ group was a physical therapy student with no experience in assisted walking. The PILT_N_ was performed in pairs of two students, alternating between contact providers and receivers. PILT was applied through the fingertips of the contact provider and was performed over the clothes of the contact receiver. The contact provider walked behind and to the right of the contact receiver either providing (PILT_E_ and PILT_N_ conditions) or not providing (NT condition) LT.

This study was conducted in a well-lit hallway of the facility. Two 10-m walking tests were performed for each condition. All three conditions were tested randomly on the same day with rest periods in between. The participants were instructed to walk 16 m naturally at a normal speed or their own pace. The contact provider was instructed to maintain contact force and lightly control it at ≤ 1 N force to avoid exceeding the force limit. The contact receiver was instructed to walk normally without consciously reacting to the contact from the contact provider as if no contact were administered. The PILT_N_ group performed one practice session prior to measurement, in which we confirmed that the fingertip pressure was ≤ 1 N on the measurement screen. Contact providers who exceeded 1 N during practice were required to continue practicing until they reached ≤ 1 N. However, no contact providers performed more than two practice sessions. This study measured the average walking speed between the midpoint and 10 m using a stopwatch operated by a third party. The average of two trials was calculated for each condition.

### Passive interpersonal light touch method

The PILT was evaluated based on the fingertip contact force and direction of movement using a compact high-precision FFS (20 mm × 20 mm × 5 mm; USL-06–50 N, TEC Gihan, Kyoto, Japan). This device measured the three components of translational force, namely, Fx, Fy, and Fz generated on the contact surface. The FFS was wirelessly connected to a portable data logger (PDL-06-SA; TEC Gihan, Kyoto, Japan). Tapes were used to fix the FFS and sensor cable to the left middle fingertip and wrist, respectively, of the provider to prevent misalignment (Figure S1). To accurately capture the pressure of the fingertip, a 12-mm diameter plate was attached to the FFS. The plate was installed facing the fingertip side. The body control responses of the contact receivers were measured for each condition using IMUs (IMS-SD, Tech Gihan, Kyoto, Japan) capable of measuring triaxial acceleration. These were placed on the seventh cervical spinous process and third lumbar spinous process. Output from the FFS and two IMUs were measured synchronously using a sensor measurement system (Tech Gihan, Kyoto, Japan). The sampling frequency of each sensor was set to 1 kHz.

**Fig. 1:**
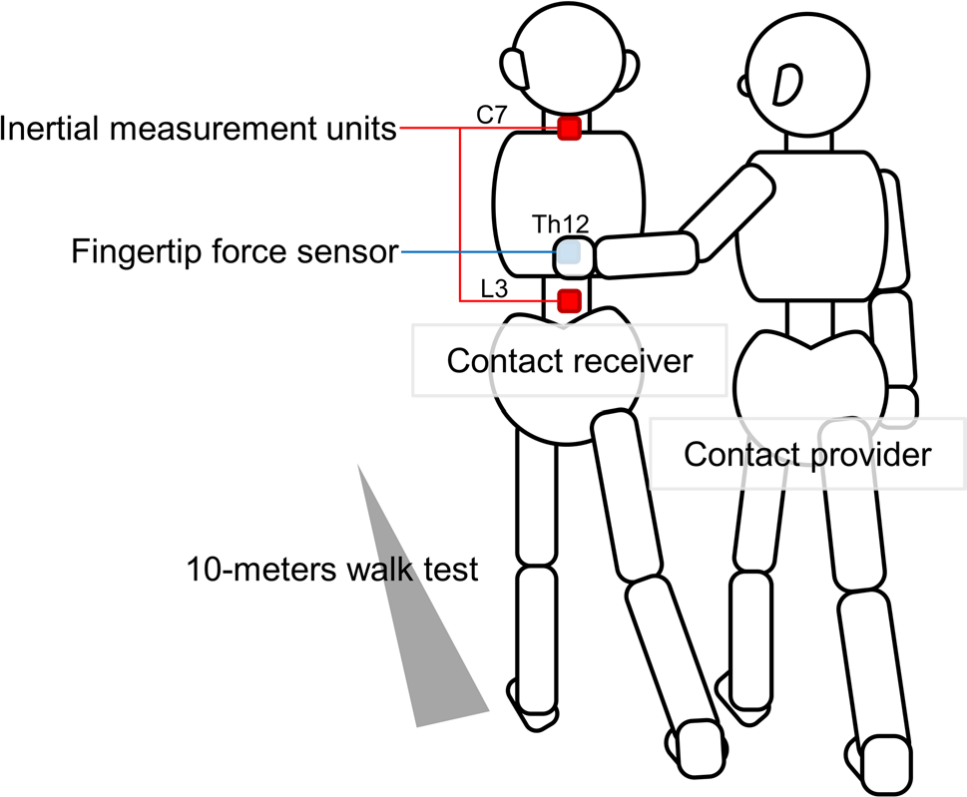
Passive interpersonal light touch method The inertial measurement units were placed on the 7th cervical spinous process and 3rd lumbar spinous process of the contact receiver. The force sensor was placed on the middle finger phalanges of the contact provider. The 10-m walk test was performed with the contact provider touching the contact receiver at the level of the 12th thoracic spinous process. Participants were instructed not to intentionally control their posture while walking

Under the PILT_E_ and PILT_N_ conditions, the left middle finger of the contact provider touched the level of the twelfth thoracic spinous process of the participant from the beginning to end of the walking process (Fig. 1). The left upper limb of the provider was positioned with mild shoulder flexion and abduction (approximately 5–15°) and external rotation of approximately 45°, and the elbow was flexed according to the participant’s height, as previously described [3].

### Sensor data analysis

Data analyses were performed using NumPy [9] and SciPy [10] packages implemented in Python 3.12. The data obtained from the FFS and IMUs were downsampled from 1 to 200 Hz. Time–series data from each IMU were filtered using a second-order Butterworth filter (zero phase) with a cutoff frequency of 0.5–20 Hz. We analyzed the latter of the two 10-m walk datasets. We identified heel contact using the acceleration data in the vertical direction and extracted five continuous strides after five steps.

#### Gait performance

We calculated the RMS, HR, and AC in the vertical (VT), anteroposterior (AP), and mediolateral (ML) directions to quantitatively assess walking ability.

The RMS provides the average amplitude of acceleration during walking, from which body sway information could be deduced [11–14]. The RMS in this study was extracted from the lumbar (RMS_L_) and cervical (RMS_C_) IMUs. The mean RMS values were calculated for the time–series data within the analysis interval. The RMS values were normalized by dividing them by the square of the walking speed [11]. Small RMS values indicate small IMU sway. Hence, small RMS_L_ and RMS_C_ values during walking indicate movements with little trunk sway.

HR is a measure of step-to-step smoothness and symmetry within the stride [15–19]. In this study, HR was calculated using the lumbar IMU (HR_L_). The HR is generally calculated from the ratio of the even and odd components extracted from the first 20 harmonic amplitudes using Fast Fourier Transform of the acceleration data. The HR for each stride of the VT and AP accelerations was calculated by dividing the sum of even harmonics by the sum of odd harmonics, whereas the HR for the ML acceleration was calculated by dividing the sum of odd harmonics by the sum of even harmonics [20, 21]. High HR values indicate a smooth walking pattern in all directions of movement.

AC is an indicator of regular walking [18, 22]. The acceleration information overlaps with similar data phase-shifted by the mean step and stride times. In this study, AC was calculated from the lumbar IMU (AC_L_). The first peak value of the AC function indicates the periodicity of successive steps, and the second peak indicates the periodicity of the strides. In the ML direction, the first peak shows a negative value, whereas the second shows a positive value [22]. The second peak values of all acceleration data were analyzed to confirm the periodicity of the stride. AC values ranged from 0 to 1, with 0 indicating no association and 1 indicating perfect agreement of gait period signals between adjacent strides [22].

#### Phase locking value of fingertip and body sway directions

The PLV is an indicator of phase synchronization between two waveforms [6]. In this study, the PLV was calculated between the fingertip movement direction (FFS) of the contact provider and body control response (IMU) of the receiver during walking. We calculated the FFS-lumbar IMU (PLV_L_) and FFS-neck IMU (PLV_C_) separately along the three axes. The time–series data from each sensor were filtered using a 0.5–2 Hz zero-phase bandpass filter to account for the gait period [23], and a Hilbert transform was used to estimate the instantaneous phase. Time-averaged PLV was calculated using the following equation [24]:

$$\:PLV=\:\frac{1}{N}\left|\sum\:_{n=1}^{N}{e}^{i({\theta\:}_{1}\left(n\right)-{\theta\:}_{2}(n\left)\right)}\right|$$

where, *N* indicates the number of sampled time points, and *θ*1 and *θ*2 indicate the instantaneous phase values at time *n* of the FFS and each IMU, respectively. The PLV values range from 0 to 1, where 0 indicates a random phase relationship, and 1 indicates perfect phase synchronization between the fingertip pressure-sensitive sensor signal and IMU signal.

### Statistical analysis

The data were analyzed in several steps. First, we confirmed the normality of all data using the Shapiro–Wilk test. As LT pressure was not normally distributed, we performed a nonparametric test. Wilcoxon’s signed-rank test was used to evaluate the differences between PILT_E_ and PILT_N_ for LT pressure relative to the forward direction of the FFS. Second, repeated measures one-way analysis of variance (ANOVA) was used to evaluate differences between the LT conditions (NT, PILT_E,_ and PILT_N_) for walking speed, RMS_L_ (RMS_L−VT_, RMS_L−AP_, and RMS_L−ML_), RMS_C_ (RMS_C−VT_, RMS_C−AP_, and RMS_C−ML_), HR_L_ (HR_L−VT_, HR_L−AP_, and HR_L−ML_), and AC_L_ (AC_L−VT_, AC_L−AP_, and AC_L−ML_) in the triaxial direction. A post-hoc test with Bonferroni correction was performed when main effects were found between conditions. Third, paired t-tests were used to assess the differences between PILT_E_ and PILT_N_ for the PLV_L_ (PLV_L−VT_, PLV_L−AP_, and PLV_L−ML_) and PLV_C_ (PLV_C−VT_, PLV_C−AP_, and PLV_C−ML_) in the triaxial direction. Additionally, effect sizes were evaluated based on the partial eta squared (η_p_^2^) for one-way ANOVA and Cohen’s *d* for t-tests. Finally, we performed a correlation analysis between walking speed and PLV (PLV_L−VT_, PLV_L−AP_, PLV_L−ML_, PLV_C−VT_, PLV_C−AP_, and PLV_C−ML_) for each PILT condition.

Statistical analyses were performed using IBM SPSS^®^ statistical software version 29 (IBM Corporation, Armonk, NY, USA). Statistical significance was set at *p* < 0.05.

## Results

The ages and sexes of the 17 healthy volunteers (21.4 ± 1.1 years, 9 female) who participated in this study are showed in Table S1. LT pressure was 0.79 ± 0.36 N during PILT_E_ and 0.60 ± 0.45 N during PILT_N_ with no significant differences between the two scenarios (*p* = 0.102).

**Fig. 2:**
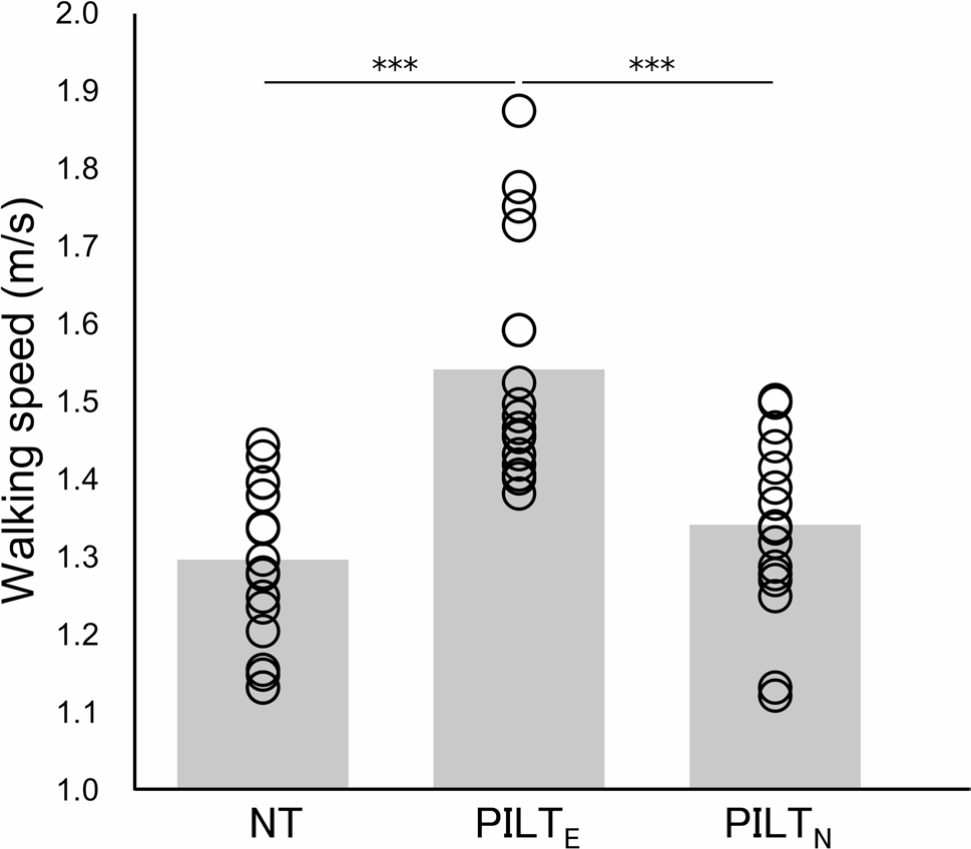
Walking speeds recorded during 10 walking tests under each light touch scenario. Black circles indicate individual values; gray bars indicate average values. NT, no touch; PILT_E_, passive interpersonal light touch administered by a contact provider with rehabilitation experience in manual body manipulation; PILT_N_, passive interpersonal light touch administered by a contact provider without rehabilitation experience in manual body manipulation

Walking speed was 1.30 ± 0.10 m/s under NT, 1.54 ± 0.15 m/s under PILT_E_, and 1.34 ± 0.11 m/s under PILT_N_. A significant main effect was identified between scenarios (*F* = 46.047, *p* < 0.001, η_p_^2^ = 0.742), and post-hoc tests showed a significant increase in speed under PILT_E_ compared with those under NT (*p* < 0.001) and PILT_N_ (*p* < 0.001) scenarios (Fig. 2).

The RMS, HR, and AC parameters for gait analysis are presented in Table 1. The walking speed and other walking parameters classified by sex are showed in Table S2. Significant main effects were identified for all RMS parameters between the LT conditions. Post-hoc tests showed significantly high values for RMS_L−VT_, RMS_L−AP_, RMS_C−VT_, RMS_C−AP_, and RMS_C−ML_ under PILT_E_ (*ps* < 0.01) than under NT and PILT_N_. PILT_E_ showed significantly high RMS_L−ML_ values than those observed during PILT_N_ (*p* = 0.009). A significant main effect was observed for HR_L−AP_ for all LT scenarios, and post-hoc tests showed that the HR_L−AP_ values were significantly higher under PILT_E_ than those under NT and PILT_N_ (*ps* < 0.05). No significant main effects were identified between the LT scenarios for other HR or AC parameters. The PLV parameters are presented in Table 2. The PLV_L−ML_ values increased significantly under PILT_E_ than under PILT_N_ (*t* = 2.167, *p* = 0.046, *d* = 0.526). In contrast, administering PILT_E_ significantly reduced the PLV_L−AP_ (*t* = − 2.442, *p* = 0.027, *d* = 0.592) and PLV_C−AP_ (*t* = − 2.498, *p* = 0.024, *d* = 0.606) values compared with those of the PILT_N_ group. The other PLV parameters did not show significant differences.

**Fig. 3:**
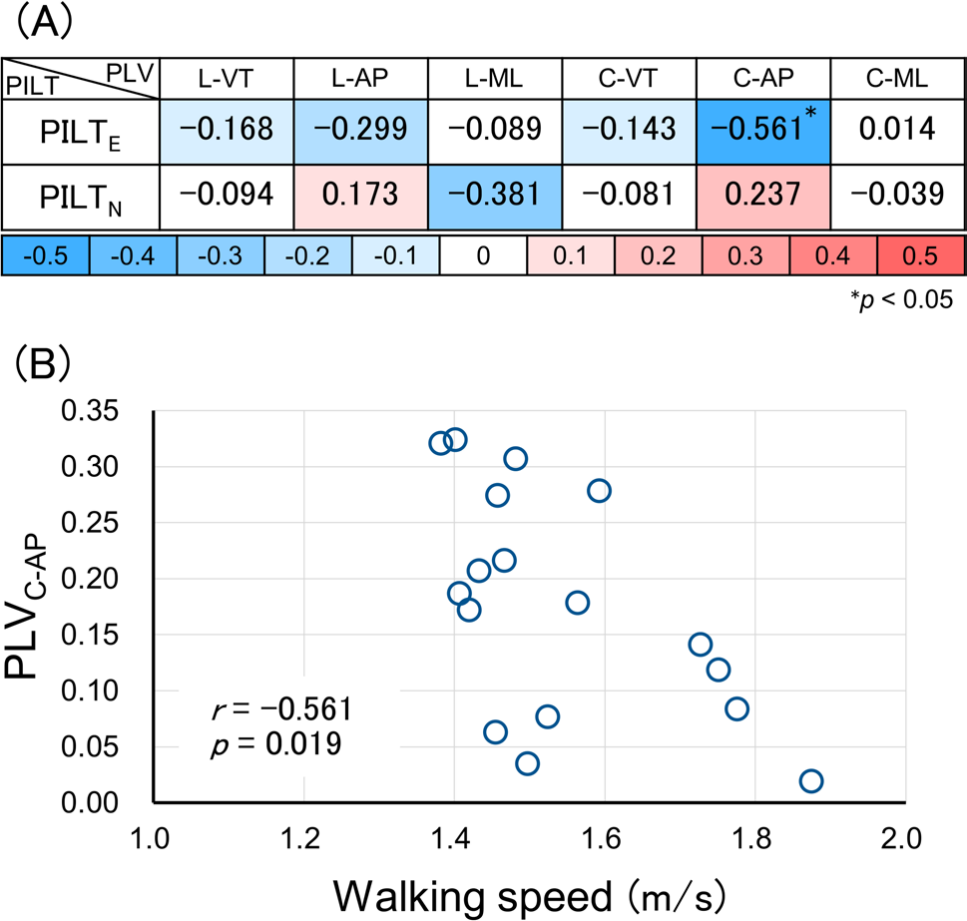
Correlations between walking speed and PLV parameters (**A**) Correlation coefficients for association between walking speed and phase locking value in each passive interpersonal light touch condition. Dark blue indicates a moderate negative correlation, and dark red indicates a moderate positive correlation. **B** Scatterplot showing the relationship between walking speed and PLV_C−AP_ during PILT_E_. WS, walking speed; PLV, phase locking value; PILT_E_, passive interpersonal light touch administered by a contact provider with rehabilitation experience in manual body manipulation; PILT_N_, passive interpersonal light touch administered by a contact provider with no rehabilitation experience in manual body manipulation; L, lumbar; C, cervical; VT, vertical direction; AP, anteroposterior direction; ML, mediolateral direction

Furthermore, walking speed showed a significant negative correlation with PLV_C−AP_ under PILT_E_ (*r* = −0.561, *p* = 0.019) (Fig. 3). No significant correlations were found with the other PLV parameters.

**Table 1:** Results of one-way analysis of variance for gait analysis parameters from all scenarios

Parameter	NT	PILT_E_	PILT_*N*_	F (df)	*P*	η_*p*_^2^
RMS_L−VT_	2.30 ± 0.33***	2.74 ± 0.56	2.36 ± 0.46***	12.106 (2,16)	< 0.001	0.431
RMS_L−AP_	1.86 ± 0.23***	2.28 ± 0.34	1.91 ± 0.38**	16.136 (2,16)	< 0.001	0.502
RMS_L−ML_	1.62 ± 0.48	1.82 ± 0.41	1.52 ± 0.55***	8.002 (2,16)	0.002	0.333
RMS_C−VT_	1.92 ± 0.28***	2.42 ± 0.37	2.04 ± 0.37**	17.459 (2,16)	< 0.001	0.522
RMS_C−AP_	1.55 ± 0.33***	1.94 ± 0.44	1.60 ± 0.34**	15.029 (2,16)	< 0.001	0.484
RMS_C−ML_	1.05 ± 0.22**	1.27 ± 0.32	1.07 ± 0.21*	10.531 (2,16)	< 0.001	0.397
HR_L−VT_	2.83 ± 2.09	4.46 ± 1.77	3.08 ± 2.26	3.042 (2,16)	0.062	0.160
HR_L−AP_	2.64 ± 2.13**	5.07 ± 2.07	3.11 ± 2.27*	6.305 (2,16)	0.005	0.283
HR_L−ML_	1.77 ± 0.73	2.46 ± 1.07	1.99 ± 0.98	3.155 (2,16)	0.056	0.165
AC_L−VT_	0.73 ± 0.04	0.74 ± 0.05	0.74 ± 0.06	1.226 (2,16)	0.307	0.071
AC_L−AP_	0.74 ± 0.03	0.75 ± 0.03	0.74 ± 0.03	0.359 (2,16)	0.701	0.022
AC_L−ML_	0.65 ± 0.06	0.68 ± 0.07	0.65 ± 0.08	1.141 (2,16)	0.332	0.067

*p < 0.05, **p < 0.01, ***p < 0.001, significant difference compared to PILT_E_ in Bonferroni test

NT, no touch; PILT_E_, passive interpersonal light touch provided by contact provider with rehabilitation experience in manual body manipulation; PILT_N_, passive interpersonal light touch provided by contact provider with no rehabilitation experience in manual body manipulation; RMS, root mean square; HR, harmonic ratio; AC, autocorrelation coefficient; L, lumbar; C, cervical; VT, vertical direction; AP, anteroposterior direction; ML, mediolateral direction

**Table 2:** PLV results between passive interpersonal light touch scenarios

Parameter	PILT_E_	PILT_*N*_	t (df)	*P*	D
PLV_L−VT_	0.28 ± 0.16	0.36 ± 0.21	−1.134 (16)	0.273	0.275
PLV_L−AP_	0.19 ± 0.08	0.31 ± 0.18*	−2.442 (16)	0.027	0.592
PLV_L−ML_	0.54 ± 0.17	0.40 ± 0.23*	2.167 (16)	0.046	0.526
PLV_C−VT_	0.26 ± 0.13	0.38 ± 0.19	−1.802 (16)	0.090	0.437
PLV_C−AP_	0.18 ± 0.10	0.33 ± 0.18*	−2.498 (16)	0.024	0.606
PLV_C−ML_	0.67 ± 0.19	0.52 ± 0.26	1.630 (16)	0.123	0.395

*p < 0.05, Significant difference compared to PILT_E_

PLV, phase locking value; PILT_E_, passive interpersonal light touch provided by contact provider with rehabilitation experience in manual body manipulation; PILT_N_, passive interpersonal light touch provided by contact provider with no rehabilitation experience in manual body manipulation; L, lumbar; C, cervical; VT, vertical direction; AP, anteroposterior direction; ML, mediolateral direction

## Discussion

This study found that administering PILT_E_ significantly increased the walking speed compared with that of NT. As tactile interactions provide cues for interpersonal postural coordination [25], the PILT provided by experts may have enhanced gait speed by constructing spatial coordinates from the fingertips to provide cues for forward propulsion. This concurs with the finding of a previous similar study [3]. Increased walking speed is associated with improved independence [26], physical activity [27], and energy efficiency [28] in patients with various diseases. Notably, the walking speed was significantly slower in the PILT_N_ group than in the PILT_E_ group with no significant difference between PILT_N_ and NT. In the PILT_N_ group, LT was provided by students with no experience in walking assistance as contact providers. Contact pressure varies depending on the contact site and posture [29]. Nevertheless, fingertip contact pressure was ≤ 1 N during both PILT_E_ and PILT_N_, and despite some variations, no significant differences were observed between the two. However, walking speed increased under PILT_E_, which was performed by expert physical therapists with experience in support activities. Thus, the physical guidance techniques for contact receivers may have affected walking speed. An additional aspect to note while interpreting the results is that this study was conducted on healthy volunteers. Participants were instructed to walk at their own pace, which suggests a possible significant potential for improvement of the walking speed. Therefore, careful consideration is required when applying these results to individuals with walking impairments.

PLV is a parameter of interpersonal postural coordination and is used in this study as an indicator of phase synchronization with respect to the directions of fingertip pressure and body sway. These findings showed that the PLV_L−ML_ value increased significantly under PILT_E_ than under PILT_N_. Contact providers who routinely assist in movement may unconsciously guide the body in the ML direction to facilitate an efficient center-of-gravity shift in the contact receiver. This study suggests that the ML direction of the contact provider’s fingertip movement, as measured during PILT_E_, moves in synchrony with the ML direction of the contact receiver’s center of gravity shift. In contrast, PILT_E_ significantly reduced the PLV_L−AP_ and PLV_C−AP_ values compared with those observed for PILT_N_. Additionally, walking speed showed a significant negative correlation with PLV_C−AP_ under the PILT_E_ scenario. Furthermore, contact providers with less experience may attempt to synchronize with changes in the AP direction, whereas more experienced providers synchronized with changes in the ML direction. This indicates that the direction of application of fingertip pressure is important for interpersonal postural coordination in PILT interventions during walking. The ML direction PLV value was higher than those of the VT and AP directions, which is supported by the correlation results between walking speed and ML direction PLV. Walking assistance provided through PILT_E_ may be influenced not just by contact stimulation and physical force but also by the recognition of spatial position information based on the contact point between the fingertips of the contact provider and the trunk of the contact receiver. Thus, walking ability may be improved by achieving body control effects in the contact receiver by presenting the appropriate direction of movement through the fingertips of the contact provider.

In the context of body-control strategies, shifting the center of gravity in the AP direction is essential for participant forward propulsion. However, this study showed results that were contrary to this hypothesis where phase synchronization of the fingertip and head movements in the AP direction reduced walking speed. The movement of the head while walking is controlled by a different mechanism than those controlling trunk movements with the head and trunk moving in different directions during the gait cycle [30]. In this study, PILT was applied in contact with the trunk. Therefore, the synchronized movement of the trunk and head may deviate from normal gait. In contrast, PILT_N_ may have increased the AP direction PLV owing to a decrease in walking speed. PILT_E_ is likely to induce body movement by moving the fingertips of the contact provider slightly faster than the body sway of the contact receiver. However, the PILT_N_ may move the contact provider’s fingertips in response to the contact receiver’s body movements. Future studies are required to analyze the timing of phase synchronization.

Regarding postural control responses to PILT, HR_L−AP_ value increased significantly under PILT_E_ than under NT. HR is an indicator of regularity and smoothness of gait patterns [17, 18]. Walking movements gain forward propulsion by repeatedly accelerating and decelerating slightly [31]. As high HR in the AP direction enables smooth forward propulsion, PILT administered by skilled individuals may contribute to efficient walking movements. In contrast, PILT_N_ significantly reduced HR_L−AP_ compared with that observed for PILT_E_; moreover, walking speed was not significantly different from that during NT. This suggests that PILT skills may affect gait stability.

Among the other IMU parameters, PILT_E_ significantly increased all lumbar and neck RMS values compared with those observed for NT. This finding is contrary to the result of a previous study, which showed that walking PILT reduced the head RMS value [3], although, in that study, the contact provider made contact over the third lumbar spinous process, whereas contact was made over the twelfth thoracic spinous process in the current study. Interpersonal postural coordination is constructed in terms of spatial coordinates based on the LT contact site [4]. Given that postural stability changes as the LT contact site changes [29], the contact site may have affected the RMS values. Our findings suggest that PILT increases walking speed but may additionally increase body sway. Further investigation is needed to determine the applicability of PILT to patients with high fall-risk or walking impairment. Additionally, the AC values were not significantly different between the three LT scenarios. This could be attributed to the fact that this study included healthy participants. As healthy participants have a regular gait, the AC values may not have changed owing to the ceiling effect. The AC index varies depending on complex walking conditions [32], and it decreases in patients with asymmetrical motor function such as patients with stroke [33]. Therefore, the effect of PILT on AC may differ in difficult walking tasks and in patients with balance disorders.

This study has several limitations. First, we did not verify the relevance of the sensor analysis results. The results of this study suggest that synchrony in the ML direction may have led to increased regularity in the AP direction owing to HR, which may have increased walking speed. The PILT effect may also vary depending on sex and gender. Particularly, the social construct of gender and physical contact between participants may be factors that influence the PILT effect. Future studies should consider statistical analysis using structural equation modeling such as path analysis and covariance structure analysis and include the sex and gender of the participants. Second, this study did not blind the contact providers to the purpose of the study. Hence, the contact providers may have intentionally attempted to stabilize the physical sway of the contact receivers. Therefore, future studies should be blinded to avoid interference from the contact provider’s intentions. Finally, this study analyzed the data solely based on walking speed and did not guarantee that walking stability was equivalent among participants. Stride length and single/double limb support times may influence the sensor data measured in this study. A previous study had proposed a method of adjusting walking speed to an equivalent stability threshold to reduce inter-individual differences in walking stability [34]. Future PILT studies should analyze data with consideration of the inter-individual differences in the target population.

Despite these limitations, this study shows that PILT_E_ may synchronize the center of gravity shift in the ML direction between the contact receiver and the fingertips of the contact provider, which may affect walking speed and contrast movements between steps in the AP direction. Additionally, this study suggests that body control responses may change according to the contact provider’s PILT skill level. These findings show the clinical significance of quality rehabilitation and assistive behavior by quantifying a touching skill that may be potentially used in these scenarios. PILT is advantageous because additional gait practice may be easily provided without the need for special equipment. This is the first study to objectively demonstrate fingertip–body synchronization using PLV and provide valuable information for establishing the significance of LT techniques.

## Conclusions

This study investigated the body control responses of contact receivers using walking PILT. Additionally, we analyzed the synchrony between the direction of movement of the contact provider’s fingertip and contact receiver’s body in the context of the contact provider’s experience. Evaluation of walking speed and AP direction has shown that more experience in PILT walking assistance may help the contact receiver to achieve smoother walking performance. Contact administered by experienced contact providers enabled higher synchrony in the ML direction at the provider’s fingertips and the receiver’s trunk compared with that observed for contact administered by less experienced providers. In contrast, the scenarios with less experienced contact providers showed higher synchrony in the AP direction than when the contact providers were more experienced. These findings objectively quantify touch skills. Overall, this study may provide valuable information on the variation in PILT effects depending on the contact provider.

## Supplementary Information

Supplementary Material 1

Supplementary Material 2

## Data Availability

The datasets generated and/or analyzed during this study are not publicly available because of their secondary use for the author’s next study, but they are available from the corresponding author on reasonable request.

## Abbreviations

LT: Light touch
PILT: Passive interpersonal light touch
FFS: Fingertip force sensor
IMU: Inertial measurement units
RMS: Root mean square
HR: Harmonic ratio
AC: Autocorrelation coefficient
PLV: Phase locking value
NT: No touch
PILT_E_: Passive interpersonal light touch by a contact provider with experience in manual body manipulation
PILT_N_: Passive interpersonal light touch by a contact provider with no experience in manual body manipulation
L: Lumbar
C: Cervical
VT: Vertical direction
AP: Anteroposterior direction
ML: Mediolateral direction
