# Supplementary material for: Synchronization of passive interpersonal light touch and body control responses during walking
Source: J Neuroeng Rehabil. 2025 Dec 3;22:258. doi: 10.1186/s12984-025-01799-2 (PMC12676807; doi:10.1186/s12984-025-01799-2)
Supplement: Supplementary file 1 — Supplementary Material 1 [file 12984_2025_1799_MOESM1_ESM.pdf]

Synchronization of passive interpersonal light touch and body control responses during walking

Tsubasa Mitsutake [1,*], Hisato Nakazono [2], Takanori Taniguchi [3], Hisayoshi Yoshizuka [3], Maiko Sakamoto [4]

1. Clinical Research Center, Saga University Hospital, 5-1-1 Nabeshima, Saga, 849-8501, Japan
2. Department of Occupational Therapy, Faculty of Medical Science, Fukuoka International University of Health and Welfare, 3-6-40 Momochihama, Sawara-ku, Fukuoka 814-0001, Japan
3. Department of Physical Therapy, Faculty of Medical Science, Fukuoka International University of Health and Welfare, 3-6-40 Momochihama, Sawara-ku, Fukuoka 814-0001, Japan
4. Education and Research Centre for Community Medicine, Faculty of Medicine, Saga University, 5-1-1 Nabeshima, Saga, 849-8501, Japan

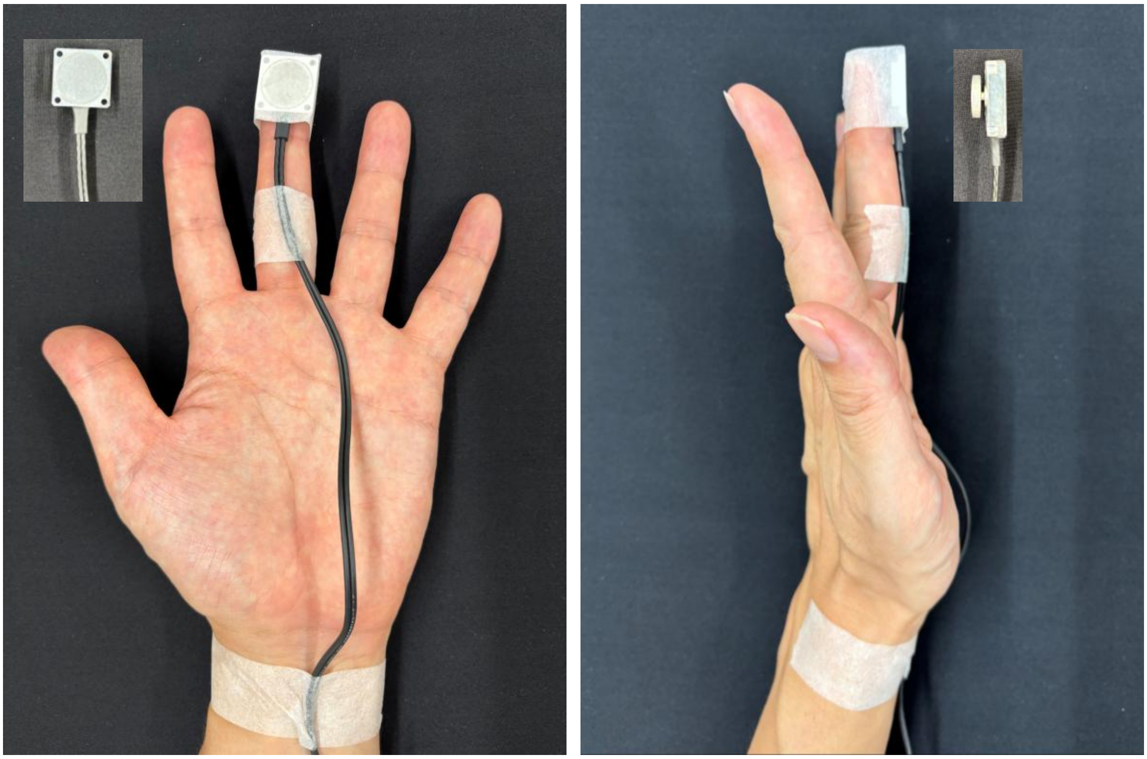

Figure S1. Showing the installation of the fingertip force sensor. A 12 mm diameter plate was attached to the FFS to accurately capture the pressure of the fingertip. The plate was installed facing the fingertip.
