# Supplementary material for: Synchronization of passive interpersonal light touch and body control responses during walking
Source: J Neuroeng Rehabil. 2025 Dec 3;22:258. doi: 10.1186/s12984-025-01799-2 (PMC12676807; doi:10.1186/s12984-025-01799-2)
Supplement: Supplementary file 2 — Supplementary Material 2 [file 12984_2025_1799_MOESM2_ESM.pdf]

Synchronization of passive interpersonal light touch and body control responses during walking

Tsubasa Mitsutake [1,*], Hisato Nakazono [2], Takanori Taniguchi [3], Hisayoshi Yoshizuka [3], Maiko Sakamoto [4]

[1] Clinical Research Center, Saga University Hospital, 5-1-1 Nabeshima, Saga, 849-8501, Japan
[2] Department of Occupational Therapy, Faculty of Medical Science, Fukuoka International University of Health and Welfare, 3-6-40 Momochihama, Sawara-ku, Fukuoka 814-0001, Japan
[3] Department of Physical Therapy, Faculty of Medical Science, Fukuoka International University of Health and Welfare, 3-6-40 Momochihama, Sawara-ku, Fukuoka 814-0001, Japan
[4] Education and Research Centre for Community Medicine, Faculty of Medicine, Saga University, 5-1-1 Nabeshima, Saga, 849-8501, Japan

Table S1. Demographic data of this study

No	Age	Sex
1	20	male
2	20	male
3	19	male
4	20	male
5	20	male
6	20	female
7	20	male
8	20	male
9	20	female
10	20	female
11	20	female
12	20	female
13	24	male
14	20	female
15	21	female
16	21	female
17	21	female
	20.4±1.1	male: 8, female: 9

Table S2. Walking speed and walking parameters according to sex						
Parameter	NT		$PILT_E$		$PILT_N$	
	male	female	male	female	male	female
WS	1.34 ± 0.10	1.26 ± 0.08	1.61 ± 0.16	1.48 ± 0.12	1.41 ± 0.08	1.28 ± 0.10
RMS_{L-VT}	2.42 ± 0.33	2.19 ± 0.30	2.96 ± 0.51	2.54 ± 0.55	2.59 ± 0.29	2.15 ± 0.50
RMS_{L-AP}	1.88 ± 0.25	1.84 ± 0.22	2.18 ± 0.28	2.37 ± 0.38	2.00 ± 0.45	1.82 ± 0.31
RMS_{L-ML}	1.77 ± 0.63	1.48 ± 0.27	1.99 ± 0.43	1.66 ± 0.34	1.74 ± 0.70	1.32 ± 0.28
RMS_{C-VT}	2.02 ± 0.35	1.82 ± 0.18	2.53 ± 0.41	2.31 ± 0.32	2.22 ± 0.40	1.88 ± 0.27
RMS_{C-AP}	1.64 ± 0.26	1.47 ± 0.38	2.05 ± 0.37	1.83 ± 0.49	1.74 ± 0.27	1.48 ± 0.37
RMS_{C-ML}	1.16 ± 0.23	0.95 ± 0.17	1.40 ± 0.35	1.15 ± 0.25	1.18 ± 0.20	0.97 ± 0.18
HR_{L-VT}	2.77 ± 2.44	2.88 ± 1.88	4.30 ± 0.76	4.60 ± 2.39	3.08 ± 1.92	3.09 ± 2.65
HR_{L-AP}	2.18 ± 1.72	3.05 ± 2.46	4.47 ± 1.01	5.60 ± 2.64	3.49 ± 2.19	2.77 ± 2.41
HR_{L-ML}	1.33 ± 0.43	2.17 ± 0.72	2.15 ± 1.01	2.73 ± 1.10	2.11 ± 1.12	1.89 ± 0.89
AC_{L-VT}	0.71 ± 0.04	0.74 ± 0.03	0.73 ± 0.07	0.75 ± 0.04	0.72 ± 0.08	0.75 ± 0.04
AC_{L-AP}	0.73 ± 0.04	0.75 ± 0.02	0.74 ± 0.04	0.75 ± 0.02	0.74 ± 0.03	0.75 ± 0.03
AC_{L-ML}	0.63 ± 0.06	0.66 ± 0.05	0.67 ± 0.07	0.68 ± 0.06	0.64 ± 0.09	0.66 ± 0.07
PLV_{L-VT}	–	–	0.23 ± 0.17	0.32 ± 0.14	0.37 ± 0.14	0.36 ± 0.26
PLV_{L-AP}	–	–	0.13 ± 0.07	0.24 ± 0.07	0.31 ± 0.23	0.32 ± 0.12
PLV_{L-ML}	–	–	0.53 ± 0.19	0.54 ± 0.17	0.36 ± 0.25	0.44 ± 0.23
PLV_{C-VT}	–	–	0.20 ± 0.09	0.32 ± 0.15	0.37 ± 0.16	0.39 ± 0.22
PLV_{C-AP}	–	–	0.12 ± 0.09	0.23 ± 0.08	0.32 ± 0.22	0.33 ± 0.15
PLV_{C-ML}	–	–	0.63 ± 0.18	0.70 ± 0.19	0.59 ± 0.24	0.46 ± 0.28

Abbreviation: NT, no touch; $PILT_E$, passive interpersonal light touch administered by a contact provider with rehabilitation experience in manual body manipulation; $PILT_N$, passive interpersonal light touch administered by a contact provider with no rehabilitation experience in manual body manipulation; WS, walking speed; RMS, root mean square; HR, harmonic ratio; AC, autocorrelation coefficient; PLV, phase locking value; L, lumbar; C, cervical; VT, vertical direction; AP, anteroposterior direction; ML, mediolateral direction.
